# Noninvasive holographic sensor system for measuring stiffness of soft micro samples

**DOI:** 10.1117/1.JBO.30.3.036501

**Published:** 2025-03-14

**Authors:** Hasan Berkay Abdioğlu, Yağmur Işık, Merve Sevgi, Ali Anil Demircali, Ufuk Gorkem Kirabali, Gokhan Bora Esmer, Huseyin Uvet

**Affiliations:** a Yıldız Technical University, Department of Mechatronics Engineering, Istanbul, Turkey; b Yıldız Technical University, Department of Bioengineering, Istanbul, Turkey; c Imperial College London, Department of Metabolism, Digestion, and Reproduction, Faculty of Medicine, London, United Kingdom; d Enlighter Medical Device Technologies Inc., Istanbul, Turkey; e Marmara University, Department of Electrical and Electronics Engineering, Faculty of Engineering, Istanbul, Turkey

**Keywords:** cell stiffness, holographic reconstruction, acousto-holographic measurement, cancer diagnostics, mechanobiology

## Abstract

**Significance**: Measuring cell stiffness is essential in cellular biomechanics, particularly in understanding disease progression, including cancer metastasis and tissue mechanics. However, conventional techniques such as atomic force microscopy and optical stretching present limitations, including invasiveness, low throughput, and complex sample preparation. These factors restrict their applicability in dynamic and sensitive biological environments.

**Aim**: This study introduces a noninvasive holographic sensor system for evaluating the stiffness of soft microscale samples.

**Approach**: The proposed system integrates holographic imaging with acoustic stimulation using an off-axis Mach–Zehnder interferometer combined with bulk acoustic waves. This setup allows for label-free, high-throughput measurements while preserving sample integrity. The system was validated with polyacrylamide beads engineered to mimic cellular stiffness, ensuring precise and repeatable stiffness assessments.

**Results**: Measurement errors caused by spatial variations were minimized through a structured imaging approach and a calibration strategy, improving uniformity across different regions. These corrections enhanced the consistency and reliability of stiffness assessments. Experimental validation demonstrated stable stiffness measurements regardless of sample size variations. Repeatability tests further confirmed the system’s robustness, producing consistent results across multiple trials.

**Conclusion**: The findings highlight the potential of this holographic sensor system in advancing cell biomechanics research, cancer diagnostics, and mechanobiology. By offering a noninvasive, high-throughput alternative for mechanical property assessments in biological samples, this method contributes to improved characterization of cellular stiffness in biomedical applications.

## 1 Introduction

Cell stiffness, a key mechanical property of biological cells, plays a fundamental role in cellular processes such as migration, division, differentiation, and adhesion.^1^^–^^5^ This property results from the complex interplay of cytoskeletal components, the extracellular matrix, and membranous organelles.^6^ Mechanobiology, the study of cellular interactions and responses to mechanical stimuli, is essential for understanding both cell physiology and pathology.^7^ In addition, it provides insights into the mechanical properties of cells and explores the impact of these properties on cellular biology. Both internal and external mechanical forces have significant functions in regulating cellular behavior and maintaining tissue homeostasis.^8^

Cell deformation is closely related to components such as filaments and the extracellular matrix.^9^^–^^11^ Each component’s deformation reflects the diverse geometries, material properties, microstructure, and external loading conditions.^12^ Understanding single-cell mechanics can provide valuable knowledge about complex cellular processes. For example, cell stiffness, largely determined by cytoskeletal components such as actin and intermediate filaments, is closely associated with biological processes involving growth, mobility, division, and differentiation.^13^ Alterations in these cytoskeletal fibers directly affect cell mechanics. Microtubules, for instance, significantly influence cell shape, motility, and division.^14^^–^^17^ Actin filaments, composed of filamentous (F) and globular (G) proteins, are essential in processes such as endocytosis,^18^ exocytosis,^19^ and maintaining cellular mechanical stability.^20^ Among cytoskeletal elements, intermediate filaments are considered mechanical buffers within cells due to their relatively low rigidity.^21^^,^^22^

Changes in cell stiffness are strongly associated with various cellular conditions and diseases. For example, cancer cells, often characterized by altered cytoskeletal structures, exhibit lower stiffness than healthy cells, facilitating increased migration and invasiveness.^23^^,^^24^ Studies in ovarian and breast cancer have shown that decreased cell stiffness correlates with cytoskeletal remodeling, underscoring its potential as a biomarker for metastatic potential.^25^^,^^26^

Several techniques are commonly used to measure cell stiffness, each with its own advantages and limitations. Micropipette aspiration involves applying negative pressure to a cell within a glass capillary and observing its elongation.^27^ This technique provides direct measurements of cell deformation and viscoelastic properties, making it valuable for studying single-cell mechanics.^28^ In addition, it allows for controlled force application and has been widely used in investigating mechanical changes in various cell types. However, precise pressure control and skilled handling are required to ensure reproducibility as the technique may alter cell properties due to the applied mechanical stress.^29^

Optical techniques, such as optical tweezers and optical stretching, utilize photon momentum to manipulate cells.^30^ These methods are noninvasive and provide high-resolution measurements while preserving cell viability.^31^ Optical tweezers can exert highly controlled forces at the piconewton scale, allowing for precise mechanical characterization of single cells.^32^ However, the reliance on specialized equipment, including high numerical aperture (NA) lenses and stable optical alignment, increases operational complexity and limits accessibility and throughput.^33^ Furthermore, optical stretching is more suitable for suspended cells, making it less effective for adherent cell populations commonly studied in mechanobiology.^34^

Atomic force microscopy (AFM) measures cell stiffness by indenting a cell with a calibrated AFM tip and analyzing the resultant force.^35^ This method offers high spatial resolution, enabling detailed subcellular mapping of mechanical properties across the cell membrane.^36^ AFM is particularly useful for detecting biomechanical changes in diseased cells, such as those associated with cancer and fibrosis.^37^ However, despite its precision, AFM is a slow technique that requires meticulous calibration and careful probe selection as variations in probe geometry, indentation depth, and substrate effects can introduce measurement inconsistencies.^38^ Moreover, the physical contact between the AFM probe and the cell membrane can lead to artifacts and may affect the mechanical properties being measured, limiting its applicability in high-throughput studies.^39^

The development of acoustic-holographic measurement in 2022 sought to combine optical and acoustic techniques for a noncontact, rapid, high-resolution approach to cell stiffness measurement.^40^ However, this approach has encountered limitations, including deviations in acoustic stimulation sources and nonuniform pressure distribution. The in-line phase-shifting holography setup, which assumes perfect sinusoidal vibrations, can also introduce distortions when actual vibration patterns vary.

**Fig. 1:**
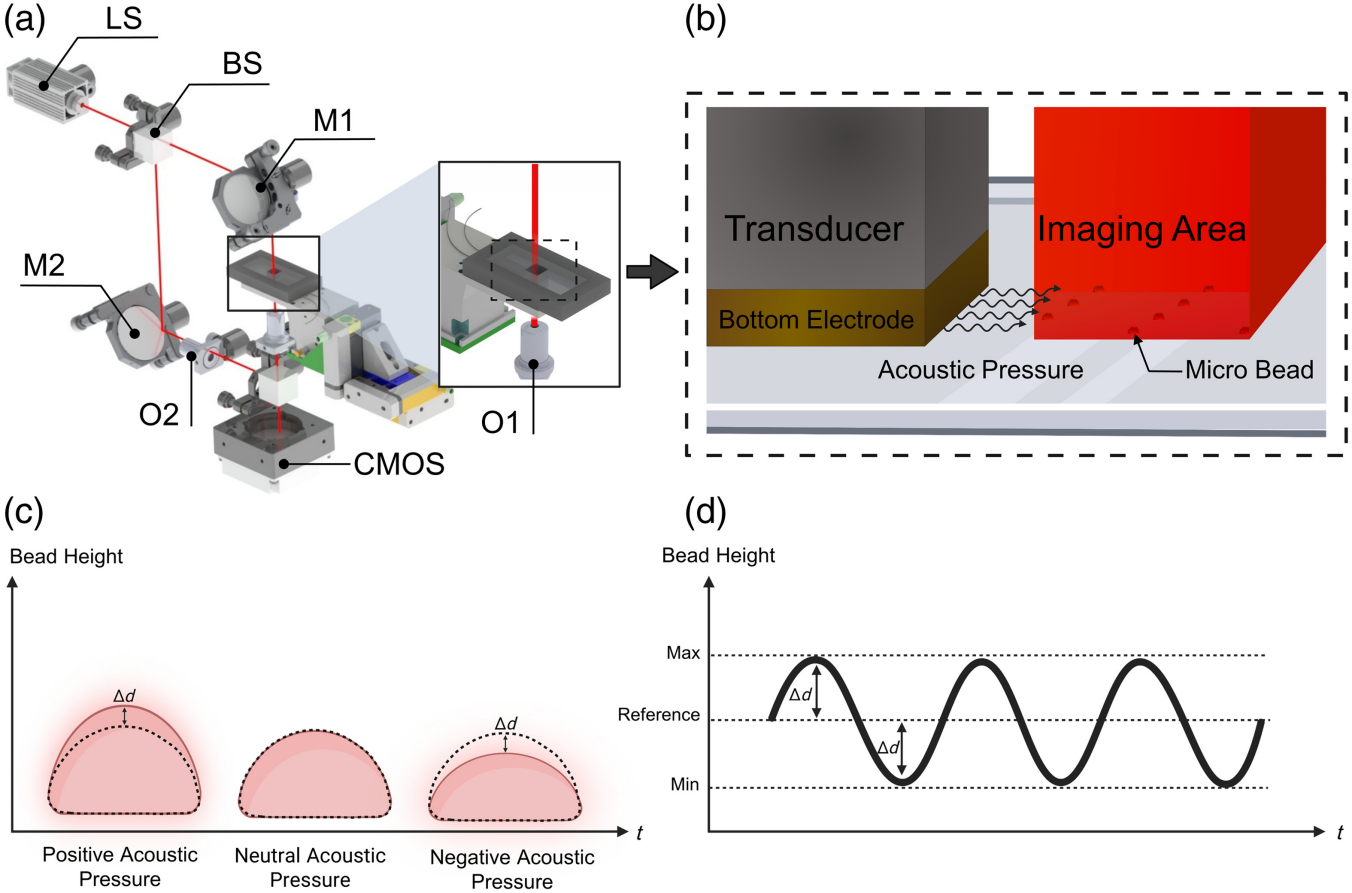
Operational principles of the stiffness measurement system. (a) Mach–Zehnder interferometric setup: illustration of a 3D model of the Mach–Zehnder interferometer used for holographic imaging. A laser beam originating from the laser source is divided into two paths by a beam splitter. The upper path transmits the beam through the object under study, whereas the lower beam remains unchanged. The beams are subsequently merged by a second beam splitter to produce an interferogram. (LS, laser source; BS, beam splitter; M1, M2, mirror; O1, O2, objective lens) (b) Chip and transducer schematic: schematic depiction of a chip integrated with a piezoelectric (PZT) transducer, surrounded by a solid enclosure and mounted on a three-axis stage for precise manipulations. The transducer is activated by signals from a wave generator, inducing acoustic pressures on the objects within the chip. (c) Thickness variation graph: graph representing the time-dependent changes in thickness value resulting from acoustic pressures. The frequency of the cell vibrations matches that of the transducer, with continuous recording via a holographic microscope to monitor the dynamic responses. (d) Sinusoidal thickness change: A graph showing the pixel-level sinusoidal variation in thickness, illustrating the detailed modulation of dimensions in response to the applied acoustic stimuli.

This study addresses the need for advanced, noninvasive, and high-throughput techniques to measure stiffness in soft micro samples, a critical parameter for both clinical diagnostics and mechanobiology research. By introducing a new hybrid sensor system based on an off-axis Mach–Zehnder microscopic setup [Fig. 1(a)], we enable observation of mechanical responses under acoustic stimulation. The system integrates a precisely designed chip with a piezoelectric transducer [Fig. 1(b)], ensuring accurate and reliable measurements through optimized acoustic conditions. Continuous holographic imaging captures time-dependent thickness variations [Fig. 1(c)], whereas pixel-level sinusoidal responses [Fig. 1(d)] reveal detailed dynamic modulations. By emphasizing appropriate vibration and sampling frequencies, as well as localization-dependent analysis, the system ensures an accurate assessment of stiffness with high precision and throughput. This noninvasive, label-free approach offers a robust solution for stiffness measurement, applicable in both research and potential clinical settings.

## 2 Methods and Materials

### 2.1 Production of Transducer-Integrated PDMS Chip

For preliminary stiffness measurements, we developed transducer-integrated poly(dimethylsil-oxane) (PDMS) chips, specifically designed to assess the mechanical properties of soft micro samples. Polyacrylamide beads, which approximate the stiffness of biological cells, were utilized as model samples to evaluate the system’s accuracy and reliability. The chip was fabricated via soft lithography, with bead fixation achieved using CELL-TAK to simulate conditions similar to those encountered by adherent cells.

The design process of the PDMS chips began with using Solidworks, followed by mold creation through aluminum milling. The microfluidic chip, with dimensions of 24  mm×60  mm×10  mm (length, width, height), was cast in PDMS using soft lithography. A mixture of silicone elastomer and curing agent at a 10:1 ratio was poured into the mold. To remove air bubbles, the mixture was placed in a desiccator for 45 min and then cured at 65°C for 3 h. Once solidified, the PDMS structure was removed from the mold with a scalpel. Finally, the PDMS structure was bonded with the transducer by applying oxygen plasma at 600 mTorr for 50 s.

### 2.2 Experimental Setup

The experimental setup was organized into two distinct subsystems: the first one for imaging, which captures three-dimensional vibration data using holography, and the second one involving the chip with an integrated transducer to generate controlled vibrations in the sample. For precise imaging and adjustment of focus across the chip, a three-axis stage (PI M-126 Precision Microtranslation Stage) was employed.

**Fig. 2:**
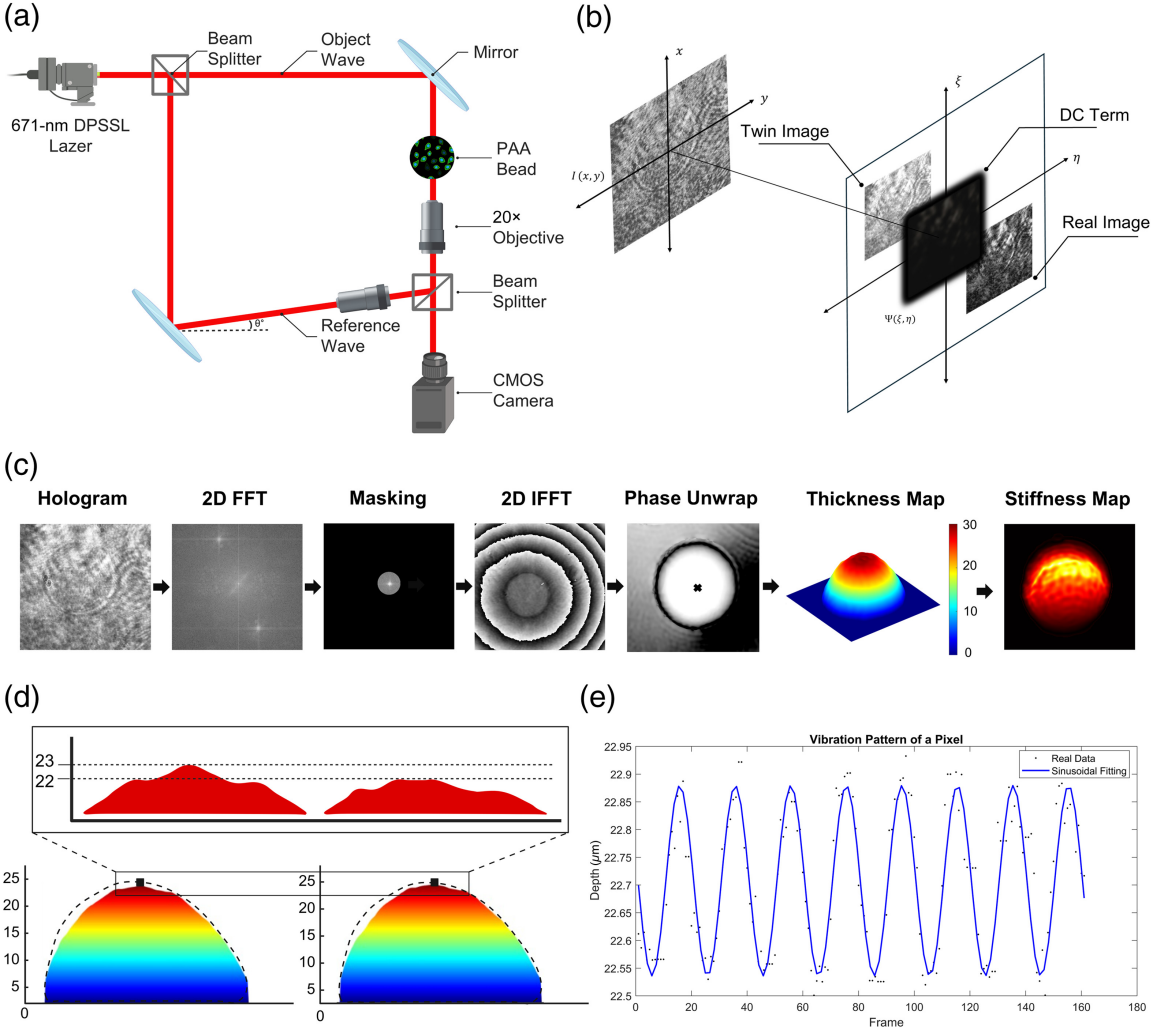
Schematics of holographic image acquisition and processing for thickness and stiffness maps: (a) A coherent 671-nm red laser is split into two beams: an object beam passing through the sample and a reference beam. The beams are recombined with a slight angle, creating interferograms that capture phase differences. (b) Interferograms contain three components (real image, twin image, DC term), which when viewed in the Fourier plane, are spatially separated due to the reference angle. (c) Interferogram processing includes recording, Fourier transformation, masking, inverse Fourier transformation, and phase extraction/unwrapping to form depth maps. Changes in these maps yield stiffness data. (d) 3D modeling of two images during microbead vibration shows dynamic responses at minimum and maximum acoustic pressures. (e) Depth changes for a chosen pixel are graphed over time, fitting a 10-Hz sinusoidal wave to match the wave generator’s frequency, confirming the vibration pattern. (Video 1 MP4, 13.6 MB [URL: https://doi.org/10.1117/1.JBO.30.3.036501.s1])

The holographic setup is based on an off-axis Mach–Zehnder interferometer, allowing rapid phase extraction by adjusting the reference beam angle [Fig. 2(a)]. This setup includes a 671-nm wavelength, 200-mW laser, two beam splitters, two mirrors, and two 20× 0.40 NA objectives. Interferogram records are captured at high speed (200 fps) using a Basler boost boA1936-400 cm CMOS camera with a 4.5-μm pixel size. A neutral density filter attenuates the laser’s intensity to optimize image clarity.

The transducer-integrated chip is securely clamped to minimize minor shifts and ensure uniform vibration distribution across the chip’s surface. Upon securing the chip, signals from a wave generator are transmitted to the transducer, initiating mechanical stimulation for data acquisition.

### 2.3 Processing of Images

#### 2.3.1 Holographic reconstruction

Three-dimensional holographic images are generated by processing off-axis hologram recordings captured with a Mach–Zehnder interferometer [Fig. 2(c)]. As illustrated in Fig. 2(a), the laser beam is split into two: the object beam passes through the sample, whereas the reference beam remains unaltered. The beams are then recombined using a beam splitter to form an interferogram.

The intensity of the captured images in the Mach–Zehnder interferometer–based microscope setup is expressed as

$$I=|\psi_{r}{|}^{2}+|\psi_{o}{|}^{2}+\psi_{r}^{*}\psi_{o}+\psi_{r}\psi_{o}^{*},$$ (1)

where $\psi_{r}$ represents the reference wave, and $\psi_{o}$ represents the object wave. The first two terms are constant (DC terms), whereas the third and fourth terms correspond to the −1st and +1st order terms, respectively. The fourth term, containing both amplitude and phase information of the object, is used for depth-related data.

To isolate this information, the hologram is filtered in the Fourier domain [Fig. 2(c)]. As shown in Fig. 2(b), the Fourier plane reveals three distinct regions: one with DC and cross terms, one with −1st order terms, and one with +1st order terms, the latter of which represents the real image.

#### 2.3.2 Reconstruction of stiffness maps

Using the acoustic-holographic setup, cell stiffness measurement is achieved through the integration of acoustic and optical systems. In this method, surface acoustic waves in the sample medium are generated using a lead zirconate titanate (PbZrTi) transducer, creating a mechanical stimulus that allows observation of the sample’s responses, as shown in Fig. 2(d). These responses are recorded using digital holographic microscopy (DHM), which combines an off-axis Mach–Zehnder interferometer with a high-speed complementary metal-oxide-semiconductor (CMOS) camera.

As surface acoustic waves propagate through the medium, they induce mechanical changes in the sample. These changes are recorded as interferograms using DHM [Fig. 2(c)]. Interferograms result from the interference of light waves from the interferometer’s reference and object beams, illustrating phase changes in the object beam relative to the reference. The obtained images provide detailed information on thickness and morphological changes induced by acoustic waves.

Each captured frame corresponds to specific vibration points, allowing the creation of vibration patterns by sequentially processing interferograms [Fig. 2(c)]. By analyzing these images, thickness maps are generated, and sample vibrations are observed [Fig. 2(d)], providing a visual representation of morphological changes.

To further validate the vibration patterns, depth changes for a chosen pixel are graphed over time, fitting a 10-Hz sinusoidal wave to match the wave generator’s frequency, as shown in Fig. 2(e). This confirms the consistency and accuracy of the vibration measurements.

To calculate sample stiffness, the Hertz elasticity model is applied, assuming samples behave as isotropic, linear elastic solids occupying an infinite half-space. The relationship between acoustic pressure and sample vibration is critical for determining stiffness, which is calculated as follows.

The displacement d at the point of vibration, obtained from holographic images, and the rest displacement $h_{0}$, can be calculated as

$$d=h-h_{0}.$$ (2)

The strain ϵ is then calculated as

$$\epsilon =\frac{d}{h_{0}}.$$ (3)

Finally, the elastic modulus E (stiffness) for each pixel j is defined as the ratio of the stress exerted on the object ($\Pi_{j}$) to the corresponding strain (ϵ)

$$E_{j}=\frac{\Pi_{j}}{\epsilon }.$$ (4)

## 3 Results

**Fig. 3:**
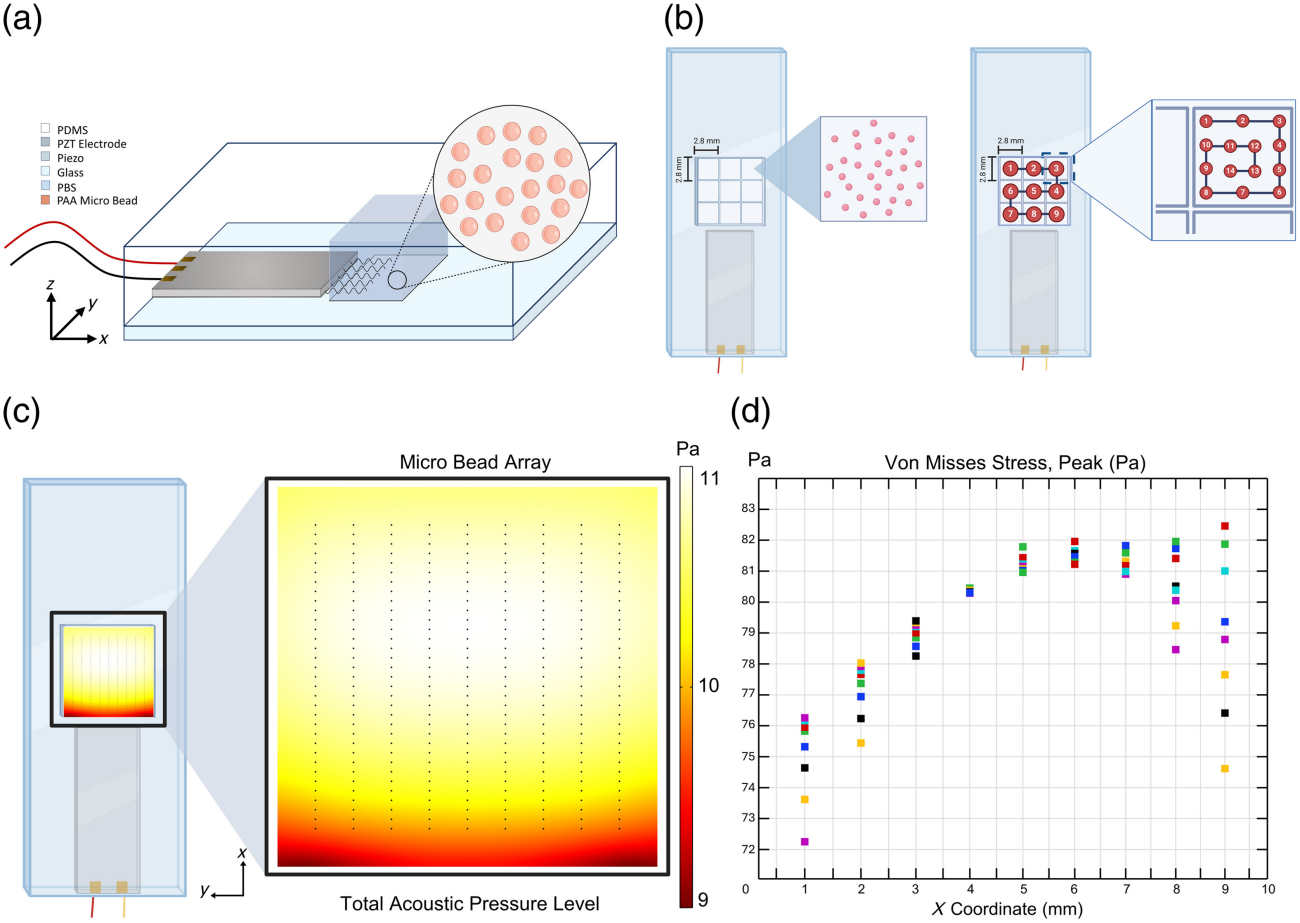
Collection of experimental measurements. (a) Structure of the chip integrated with a transducer: This part depicts the setup where microbeads are fixed on a square plane measuring $10\times 10\;{\mathrm{mm}}^{2}$. The vibration of the microbeads is induced by applying a voltage to the transducer. For each bead measurement, it is required that the three-axis stage remains stationary for 15 s. (b) Sequential image acquisition from each bead: After capturing the image from one bead, the stage moves sequentially to the next region. This transition requires the stage to progress orderly and ensures that at least one single bead is present in each region. To achieve a homogeneous distribution of measurements, navigation is conducted with equal scaling, and images are collected across nine equal square divisions, each containing data from 14 different regions, and the stiffness measurements are analyzed accordingly. (c) COMSOL simulations of acoustic pressure distribution: The result of the COMSOL simulations showing the acoustic pressure distribution across a $10\times 10\;{\mathrm{mm}}^{2}$ area. The image on the left shows a top view, whereas the image on the right displays the distribution from a side view. (d) Distribution of stress on microbeads according to COMSOL simulation results based on the acoustic pressure distribution on the chip.

The chip design of the sensor system [Fig. 3(a)] was guided by comprehensive simulations conducted in COMSOL Multiphysics 6.0, which explored a range of frequencies and sample sizes (see Figs. S1 and S2 in the Supplementary Material). The system was designed to optimize the usability of the experiment by selecting parameters that ensure stability and efficiency. From the simulations, lower frequencies were chosen to maintain a more stable response across the entire chip surface [Figs. 3(c) and 3(d)]. In addition, reducing the number of frames required to extract stiffness results from hologram images was prioritized. This approach minimized the sampling frequency requirement of imaging, reducing the overall size of the images and expediting image processing.

**Fig. 4:**
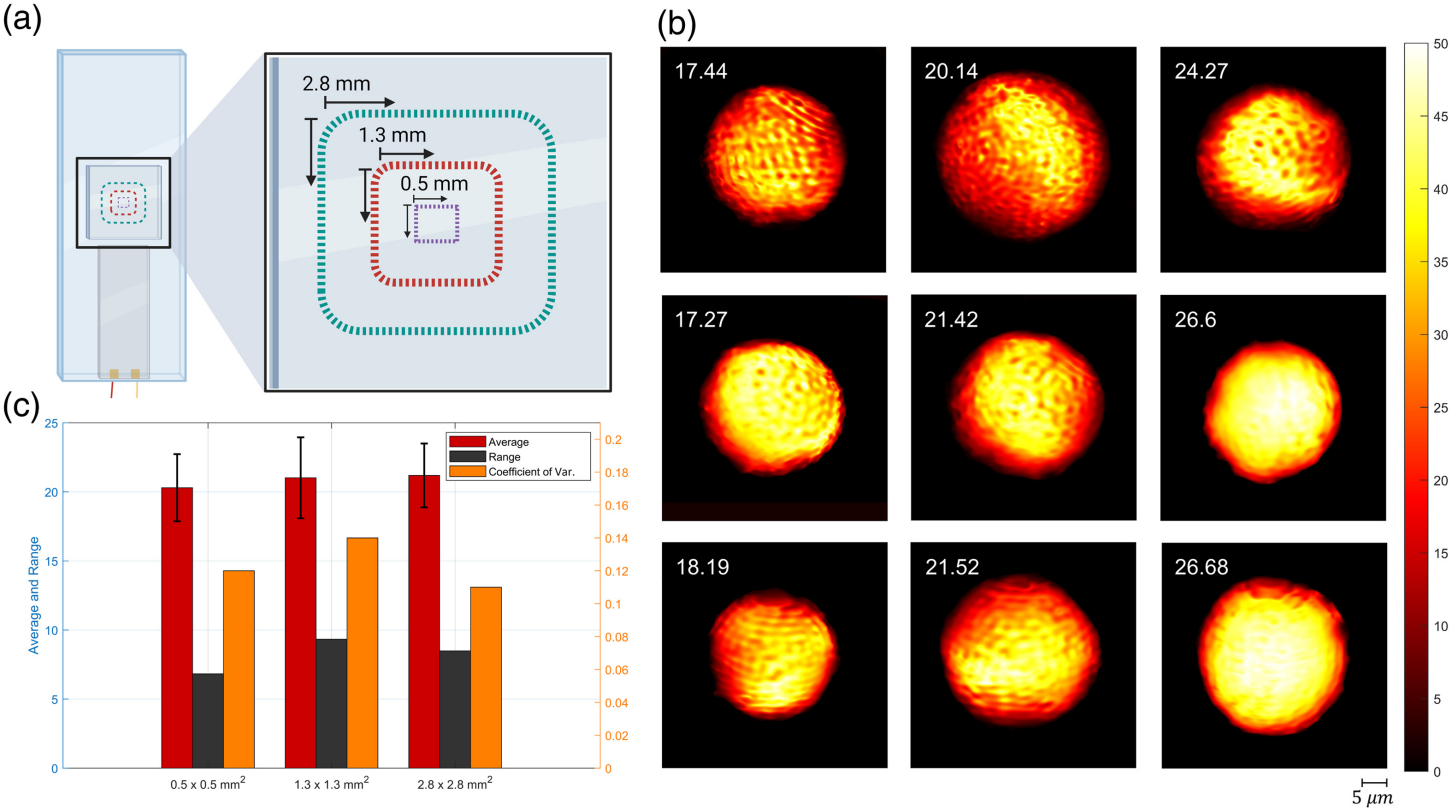
(a) 1/ϵ maps, proportional to the stiffness values of microbeads of varying sizes in the same region. The average 1/ϵ values are indicated in the top left of the maps, and the bead diameters are specified at the bottom. (b) Results of repeatability tests. Box plot of stiffness values obtained from a total of 75 different tests conducted on 10 different beads taken from the same region (square). Measurements were repeated five times on five beads and 10 times on another five beads to examine measurement repeatability and calculate deviations (Table S6 in the Supplementary Material).

The optimal configurations identified through simulations were experimentally tested to validate the system’s applicability. Polyacrylamide beads were chosen as the test material due to their stability and stiffness properties, which closely resemble those of biological cells. The sensor system was evaluated using a vibration frequency of 10 Hz and a high capture speed of 200 fps, achieving consistent and reliable stiffness measurements. The choice of a 10-Hz vibration frequency was guided by simulation results and Nyquist criteria, which also determined the optimal camera speed of 200 fps. To prevent signal overlap and ensure measurement accuracy, polyacrylamide beads (Bio-Gel PAA beads, <45  μm) were evenly distributed across the chip surface using CELL-TAK, effectively mimicking the conditions of adherent cells (Fig. 4).

**Fig. 5:**
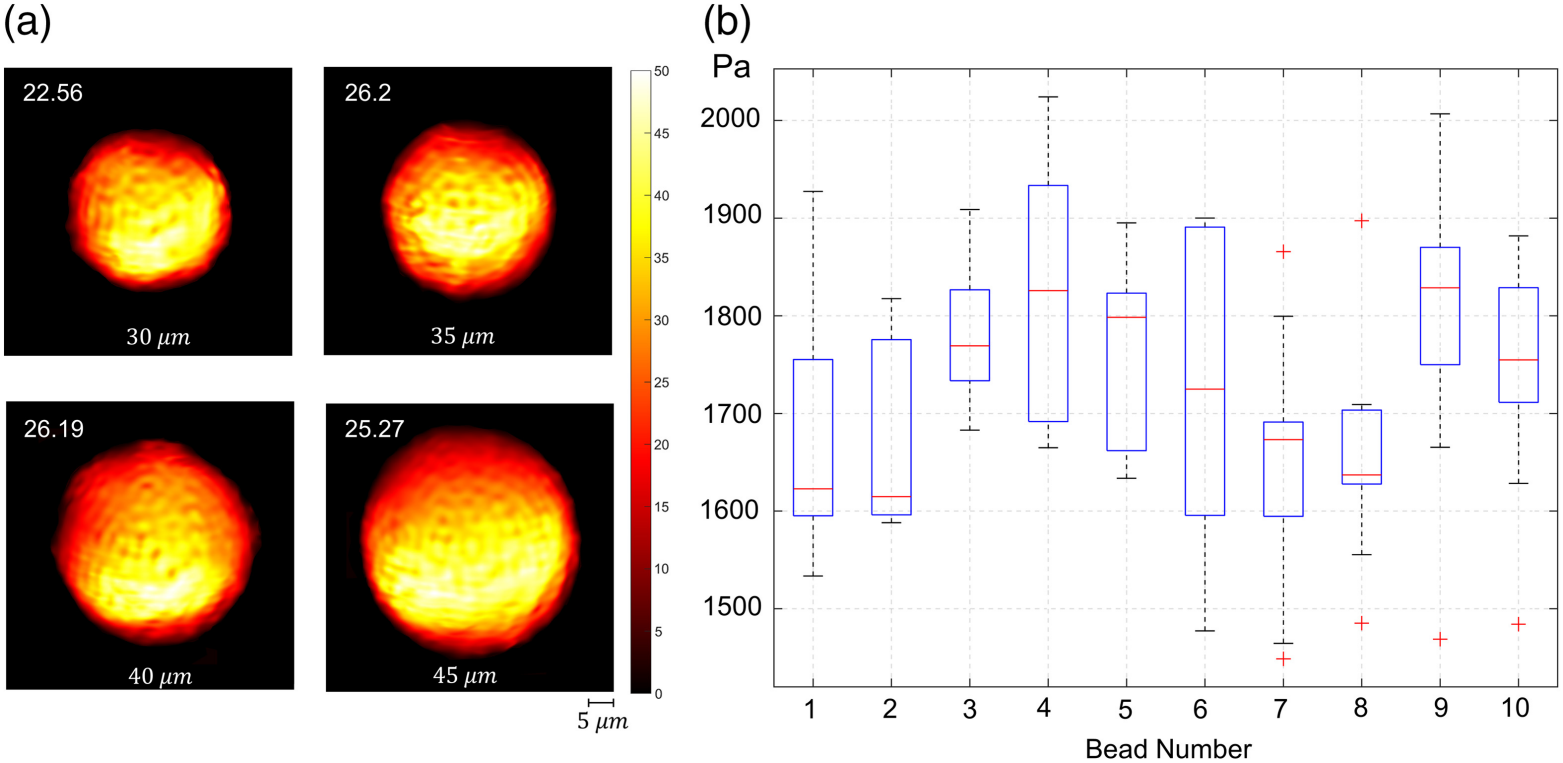
(a) Illustration of the imaging areas on the chip where measurements were taken. (b) The 2D maps of beads represent the minimum, median, and maximum 1/ϵ values obtained from the 0.5  mm×0.5  mm, 1.3  mm×1.3  mm, and 2.8  mm×2.8  mm areas, respectively. (c) Mean, standard deviation, range, and coefficient of variation values obtained from measurement areas of different sizes. Measurements were taken from 10 different beads in each area.

Repeatability tests were conducted to evaluate measurement consistency across multiple trials. The results showed a coefficient of variation value of 5.5% to 8.5%, demonstrating the sensor’s reliability in stiffness measurement, with data collected over 75 measurements from 10 distinct experiments [Fig. 5(b)]. In addition, the results indicated that bead diameter does not directly influence the resultant 1/ϵ values. Beads with diameters of 30, 35, 40, and 45  μm produced 1/ϵ values of 22.56, 26.20, 26.19, and 25.27, respectively, as depicted in Fig. 5(a). A Pearson correlation test between bead size and stiffness measurements yielded a correlation coefficient of r=0.6096 with a p-value of p=0.3904. As the p-value is greater than 0.05, the correlation is not statistically significant, indicating that variations in stiffness measurements are independent of bead size. This further confirms that bead size does not systematically affect the obtained stiffness values.

To evaluate the reliability of the imaging system in detecting object vibrations, experiments were conducted at various frequencies ranging from 10 to 100 Hz, covering 10 distinct frequency regions. At each frequency, five repeatability tests were performed, resulting in a total of 50 experiments across the 10 frequency values. The results indicate that the 1/ϵ values vary with frequency, showing a noticeable change. A sharp variation in 1/ϵ values was observed beyond 80 Hz, emphasizing a frequency-dependent behavior in the system’s measurements. Despite these changes in 1/ϵ values, the coefficient of variation values remained between 2.5% and 18.4% (Table S7 in the Supplementary Material), demonstrating that repeatability is preserved across different frequencies.

To further assess the applicability of the method to various materials, agarose beads were used in an additional repeatability experiment. For each of the five beads tested, five experiments were conducted, resulting in a coefficient of variation values ranging from 1.4% to 8.7%. These results confirm the system’s capability to accurately measure stiffness values for a wide range of soft materials (Table S8 in the Supplementary Material).

To assess the impact of imaging area size on measurement variation, tests were conducted in three different areas: 0.5  mm×0.5  mm, 1.3  mm×1.3  mm, and 3  mm×3  mm, as illustrated in Fig. 4(a). For each area size, 10 experiments were performed, revealing that standard deviation values and coefficient of variation values remained stable regardless of the area size [Fig. 4(c); Table S5 in the Supplementary Material]. Furthermore, the similarity observed among the minimum, median, and maximum 1/ϵ values across the different areas indicates consistent stiffness measurements, further validating the robustness of the system [Fig. 4(b)].

To examine location-dependent variability in greater detail, a 3×3 matrix was applied [Fig. 3(b)], dividing the 10  mm×10  mm imaging area into nine equal squares. Although standard deviation values were relatively consistent across all squares, the average stiffness values showed some variation (see Tables S1–S3 in the Supplementary Material). This observation suggests slight spatial heterogeneity in stiffness measurements within the imaging area.

To minimize variability across the chip, a square-based calibration method was implemented using a genetic algorithm, assigning a unique calibration coefficient to each square (Table S4 in the Supplementary Material). Prior to calibration, the overall coefficient of variation of the chip was 18.5%, which was reduced to 15.1% after calibration. In addition, calibration significantly improved measurement accuracy by reducing the coefficient range from 0.433 to 0.330 and the relative range from 0.871 to 0.681.

Simulation data estimated the average stress applied on the bead surfaces to be 75 Pa, resulting in an average stiffness measurement of 1828 Pa. This measurement closely aligns with findings reported by Varol et al.,^40^ further validating the sensor’s accuracy and reliability in stiffness measurements.

For applications requiring relative stiffness comparisons—such as distinguishing cancerous cells from healthy ones or assessing stiffness in metastatic cells—consistent results can be achieved by imaging samples within the same calibrated squares. Conversely, when a general stiffness distinction is sufficient, measurements taken from various regions on the chip may be adequate. This flexibility highlights the system’s adaptability for diverse biological applications, making it a robust tool for research and diagnostics.

## 4 Discussion

The proposed noninvasive holographic sensor system represents a significant advancement in cell stiffness measurement, overcoming key limitations of traditional techniques such as AFM, optical tweezers, and micropipette aspiration. Its label-free, high-throughput design ensures sample integrity, making it particularly suited for mechanobiology research and cancer diagnostics.

By differentiating malignant from healthy cells based on stiffness, this system has the potential to improve early cancer detection and disease monitoring. Its real-time, artifact-free measurements provide critical insights into cellular dynamics and mechanical responses, enhancing both diagnostic accuracy and mechanobiological studies.

The system has been tested on reference particles, assuming them to have uniform stiffness. However, actual stiffness may vary depending on the individual bead’s composition and structure. Future studies should consider material heterogeneity and assess stiffness variations across different bead types. Evaluating live cells and diverse biological samples will further validate the robustness and applicability of this approach.

This system holds transformative potential for cell biomechanics and clinical diagnostics. Future work should emphasize interdisciplinary collaboration to refine the technology and facilitate its integration into practical applications.

## 5 Conclusion

This study introduces a novel, noninvasive sensor system for accurately measuring the stiffness of soft micro samples using acoustic vibrations and holographic imaging. The system successfully generates high-resolution, label-free 2D stiffness maps, enabling precise differentiation with a sensitivity of up to 11% of the mean stiffness value in regional measurements (Table S2 in the Supplementary Material).

To achieve optimal performance which will provide repeatable and reliable results for every possible measurement, the transducer-integrated chip was first designed and integrated into the optical setup, followed by simulations to determine the most suitable chip configuration for repeatable and reliable measurements. Experimental validation was conducted using reference particles with mechanical properties similar to cellular structures. The results of this study demonstrated high repeatability, with coefficient of variation values ranging from 5.5% to 8.5% for polyacrylamide beads and 1.4% to 8.7% for agarose beads.

Further analysis of the system’s frequency response between 10 and 100 Hz confirmed measurement consistency, with coefficient of variation values observed between 2.5% and 18.4%. In addition, the spatial distribution of the acoustic pressure was examined across the chip surface, revealing that dividing the imaging area into a 3×3 matrix enhances measurement accuracy and minimizes location-dependent variations. By assigning a calibration coefficient to each square, the coefficient of range across the entire chip surface was reduced by 1.3, leading to an improvement in measurement uniformity. This calibration method effectively lowered the coefficient of range from 0.433 to 0.330, ensuring greater reliability in stiffness measurements.

Therefore, this study demonstrates the system’s potential for applications in mechanobiology, cancer diagnostics, and biomedical research, where precise stiffness differentiation is crucial. Future work will focus on further miniaturization and clinical validation, ensuring broader applicability in real-world diagnostic and research settings.

## Supplementary Material

10.1117/1.JBO.30.3.036501.s01

10.1117/1.JBO.30.3.036501.s1

## Data Availability

The data that support the findings of this study are available from the corresponding author upon request.
