# Supplementary material for: Noninvasive holographic sensor system for measuring stiffness of soft micro samples
Source: J Biomed Opt. 2025 Mar 14;30(3):036501. doi: 10.1117/1.JBO.30.3.036501 (PMC11907929; doi:10.1117/1.JBO.30.3.036501)
Supplement: Supplementary file 1 [file JBO_030_036501_SD001.pdf]

Non-Invasive Holographic Sensor System for Measuring Cell Stiffness Supplementary Information

Contents:

Supplementary Table S1: 1/ϵ values for polyacrylamide microspheres along three distinct squares taken from the chip surfaces are presented herein for the first row.

Supplementary Table S2: 1/ϵ values for different beads in the second row.

Supplementary Table S3: 1/ϵ values for different beads in the third row.

Supplementary Table S4: Calibration coefficients of 3x3 array for each row and column.

Supplementary Table S5: 1/ϵ values for different beads along squares of varying sizes

Supplementary Table S6: 1/ϵ values for repeated measurements over 10 different beads with total of 75 measurements.

Supplementary Table S7: 1/ϵ values for repeated measurements over 10 different frequencies between 10 Hz. and 100 Hz.

Supplementary Table S8: 1/ϵ values for repeated measurements over 5 different Agarose beads

Supplementary Figure S1: COMSOL Simulation of Acoustic Pressure Distribution (100 Hz - 2 kHz)

Supplementary Figure S2: COMSOL Simulation of Acoustic Pressure for Square-Shaped Imaging Areas (10, 15, 20 mm)

Supplementary Video S1: Vibration pattern of a polyacrylamide bead vibrating at 10 Hz, recorded at 500 fps. The accompanying phase-frame graph represents the displacement of a pixel at the center of the bead over time.

This supplementary document provides additional data and detailed results to support the findings presented in the manuscript titled *"Non-Invasive Holographic Sensor System for Measuring Stiffness of Soft Micro Samples."* The supplementary materials include quantitative stiffness measurements for polyacrylamide and agarose beads, experimental repeatability tests, and COMSOL simulation results. While the experiments were conducted on specific materials, the focus of this study is to demonstrate the universal applicability of the proposed system for all types of soft materials that can be vibrated using an acoustic transducer.

The data tables summarize measurements in the form of 1/ϵ values, reflecting the strain component of the elastic modulus equation. Unlike conventional stiffness measurements that calculate the elastic modulus directly, this system measures strain as part of the equation, providing high-precision and consistent results. The stress component was determined using COMSOL Multiphysics simulations, with an average value of 75 pascals for a 10 Hz application.

Supplementary Table S1: 1/ϵ values for polyacrylamide microspheres along three distinct squares taken from the chip surfaces are presented herein for the first row. Tables 1, 2, and 3 present the comprehensive stiffness measurements (1/ϵ values) obtained from all rows and columns of the designed transducer-integrated chip. These tables highlight the spatial distribution of measurements across the chip.

Bead No.	Row 1 Column 1	Row 1 Column 2	Row 1 Column 3
1	23.24	25.96	26.27
2	24.25	24.71	27.2
3	24.69	25.22	19.24
4	22.67	31.29	17.78
5	29.34	26.18	23.07
6	20.83	22.56	23.36
7	29.17	25.27	22.72
8	30.36	26.3	26.27
9	21.66	26.79	17.83
10	34.33	29.5	29.53
11	28.36	25.34	31.54
12	19.41	24.59	24.77
13	24.18	26.18	24.69
14	19.17	25.19	21.97
Average	25.12	26.07	24.02
Standard Deviation	4.53	26.08	24.02
Coef. Of Variation	0.18	0.8	0.17
Coef. Of Range	0.28	0.16	0.28

Supplementary Table S2: 1/ϵ values for different beads in the second row.

Bead No.	Row 2 Column 1	Row 2 Column 2	Row 2 Column 3
1	13.93	23.24	17.53
2	14.78	21.52	20.41
3	17.75	18.19	21.64
4	15.04	19.86	19.49
5	14.55	26.68	23.41
6	20.95	22.35	29.13
7	23.48	23.87	25.64
8	27.18	18.71	23.44
9	17.73	18.83	24.28
10	17.08	21.42	22.79
11	27.67	21.5	25.31
12	25.1	20.7	20.04
13	17.12	19.96	20.29
14	21.99	19.82	22.03
Average	19.6	21.19	22.53
Standard Deviation	4.77	2.31	2.99
Coef. Of Variation	0.24	0.11	0.13
Coef. Of Range	0.33	0.19	0.25

Supplementary Table S3: 1/ϵ values for different beads in the third row.

Bead No.	Row 3 Column 1	Row 3 Column 2	Row 3 Column 3
1	27.43	25.01	23.69
2	27.37	23.47	24.46
3	33.43	22.76	23.65
4	26.75	23.39	26.12
5	24.15	32.56	27.16
6	26.13	20.33	23.86
7	26.27	22.37	21.18
8	35.18	21.35	29.21
9	25.08	18.29	30.6
10	31.49	29.12	29.33
11	29.85	23.4	32.3
12	24.25	28.15	34.29
13	28.05	26.31	28.07
14	30.62	33.08	32.75
Average	28.29	24.97	27.62
Standard Deviation	3.38	4.39	3.98
Coef. Of Variation	0.12	0.17	0.14
Coef. Of Range	0.19	0.29	0.24

Supplementary Table S4: Calibration coefficients of 3x3 array for each row and column. Row 2 Column 2 is taken as reference for other squares. It presents the calibration coefficients derived from the data in Tables 1, 2, and 3, providing a standardized approach for measurements across the chip surface. The reference point is set at row 2, column 2, meaning measurements taken from the central square can be used directly without any correction. For measurements from other regions, the corresponding calibration coefficients must be applied to ensure consistency and comparability.

	R1C1	R1C2	R1C3	R2C1	R2C2	R2C3	R3C1	R3C2	R3C3
Calibration Coefficients	0,82	0,815	0,869	1,038	1	0,935	0,748	0,832	0,761

Supplementary Table S5: 1/ϵ values for different beads along squares of varying sizes. This analysis evaluates the impact of imaging area size on measurement accuracy and precision, highlighting the robustness of the system across different spatial scales. The results demonstrate that the random distribution of beads within the imaging areas does not introduce significant variability. When imaging a chip with randomly distributed beads, the sampled regions are effectively random parts of the area. This analysis confirms that varying the size of the imaging areas does not spatially affect the measurement outcomes, ensuring consistency and reliability regardless of the selected region size.

	$0.5\,x\,0.5$ mm^2	$1.3\,x\,1.3$ mm^2	$2.8\,x\,2.8$ mm^2
1	23.32	19.5	23.24
2	18.14	19.96	21.52
3	20.14	21.42	18.19
4	22.56	21.5	19.86
5	21.49	20.7	26.68
6	24.27	17.27	22.35

**7**	20.5	26.6	23.87
**8**	17.54	25.3	18.71
**9**	17.44	18.14	18.83
**10**	17.66	19.82	21.42
**Average**	2.42	2.93	2.3
**Standard Deviation**	20.31	21.02	21.18
**Coef. Of Variation**	0.12	0.14	0.11
**Coef. Of Range**	0.16	0.21	0.19

Supplementary Table S6: 1/ϵ values for repeated measurements over 10 different beads with total of 75 measurements. The data demonstrate consistent stiffness measurements across multiple trials, validating the system's ability to vibrate and image the beads effectively. These findings, combined with results from spatially distributed beads, indicate that the system maintains consistent vibration characteristics across the chip surface. If repeatability could not be achieved, it would point to inconsistencies in the vibration patterns or abnormal spatial effects on the beads, making the chip surface unsuitable for stiffness measurements in random locations. However, the results confirm the robustness of the system, further enhanced by a straightforward calibration technique that divides the surface into nine areas and assigns each a unique coefficient to ensure measurement accuracy.

	Bead 1	Bead 2	Bead 3	Bead 4	Bead 5	Bead 6	Bead 7	Bead 8	Bead 9	Bead 10
**Exp 1**	20.83	20.73	20.81	19.59	19.22	17.38	17.04	19.15	21.58	20.79
**Exp 2**	19.01	21.38	19.80	20.01	19.66	19.08	19.85	20.04	23.61	19.16
**Exp 3**	22.68	18.68	21.17	21.48	21.17	18.17	17.23	18.30	20.67	22.14

**Exp 4**	19.98	19.00	20.59	22.39	21.16	22.25	21.17	19.18	21.45	20.34
**Exp 5**	18.04	18.81	22.46	23.81	22.30	18.77	19.53	19.46	17.28	21.87
**Exp 6**						22.35	21.95	20.11	22.00	20.13
**Exp 7**						19.73	19.84	19.17	22.14	20.50
**Exp 8**						20.86	19.90	22.32	20.59	20.79
**Exp 9**						21.30	19.32	17.47	21.92	21.52
**Exp 10**						22.32	18.76	19.34	19.59	17.46
**Average**	20.11	19.72	20.97	21.46	20.7	20.22	19.46	19.45	21.08	20.47
**Standard Deviation**	1.59	1.11	0.87	1.55	1.12	1.75	1.45	1.2	1.63	1.3
**Coef. Of Variation**	0.08	0.06	0.04	0.07	0.05	0.09	0.07	0.06	0.08	0.06

Supplementary Table S7: $1/\epsilon$ values for repeated measurements over 10 different frequencies between 10 Hz. and 100 Hz. Table 7 displays the frequency-dependent stiffness measurements for polyacrylamide beads (PAA), highlighting the system's sensitivity to varying acoustic stimulation frequencies and its potential for dynamic mechanical testing. Beyond 100 Hz, accurately sampling the vibrations becomes challenging due to the Nyquist criterion and the limitations of the camera, which operates at a maximum of 500 fps. Consequently, the study focuses on frequencies up to 100 Hz to ensure reliable measurements. Simulations (Figure 1) demonstrate that lower frequencies provide greater stability across the chip surface, further supporting the decision to prioritize these frequency ranges for optimal performance and accuracy.

	10 Hz	20 Hz	30 Hz	40 Hz	50 Hz	60 Hz	70 Hz	80 Hz	90 Hz	100 Hz
**Exp 1**	22.69	28.44	32.67	42.68	43.66	19.16	24.48	64.35	59.87	64.75
**Exp 2**	20.89	25.38	31.6	45.32	40.38	25.47	27.64	57.57	52.69	64.67
**Exp 3**	21.86	28.41	32.18	40.57	44.26	28.75	27.27	56.3	67.45	65.33
**Exp 4**	23.81	25.42	31.19	30.96	40.08	30.79	25.68	62.87	52.11	55.22
**Exp 5**	25.05	30.03	37.22	34.33	36.61	34.02	22.65	67.32	52.56	61.31
**Average**	22.86	27.54	32.97	38.77	41	27.64	25.54	61.68	56.93	62.26
**Standart Deviation**	1.45	1.83	2.18	5.32	2.76	5.07	1.84	4.15	5.99	3.79
**Coef. Of Variation**	0.06	0.06	0.06	0.13	0.06	0.18	0.07	0.06	0.1	0.06

Supplementary Table S8: $1/\epsilon$ values for repeated measurements over 5 different Agarose beads. Focuses on experiments conducted using agarose beads, presenting repeatability results that validate the versatility of the system for assessing various types of soft materials. The material being measured must be sufficiently soft, as materials with higher elastic modulus values may not vibrate effectively with an acoustic transducer or may exhibit vibrations at nanometer scales, making it difficult to distinguish these vibrations from optical and mechanical noise disturbances. Therefore, the system is particularly well-suited for biological materials, such as cells, where accurate and reliable stiffness measurements are critical.

Bead No.	Bead 1	Bead 2	Bead 3	Bead 4	Bead 5
**Exp 1**	26.464	26.791	27.7844	24.0767	26.6001
**Exp 2**	26.1785	26.9133	26.427	28.9947	24.2839
**Exp 3**	26.5345	27.487	28.1136	30.2787	28.8511
**Exp 4**	25.4718	27.0374	27.4341	27.3405	29.2121

**Exp 5**	26.1491	27.1398	28.2674	29.6566	28.5554
**Average**	26.1595	27.0737	27.6053	28.0695	27.5005
**Standart Deviation**	0.3759	0.2375	0.6552	2.2239	1.8452
**Coef. Of Var.**	0.0143	0.0087	0.0237	0.0792	0.0671

The simulation results shown here were used to determine the optimal parameters in designing the transducer integrated chip at the beginning. Lower frequency and $10\ x\ 10\ mm^2$ imaging area is selected from the simulations, the main purpose was to design the system in a way that can be produced easily and gives reproducible, repeatable, and absolute values for differentiating different elastic modulus of biological materials. In this manner the frequency responses of chips surface is shown in figure 1 and the spatial distribution of acoustic pressure depending on imaging area size is shown in figure 2.

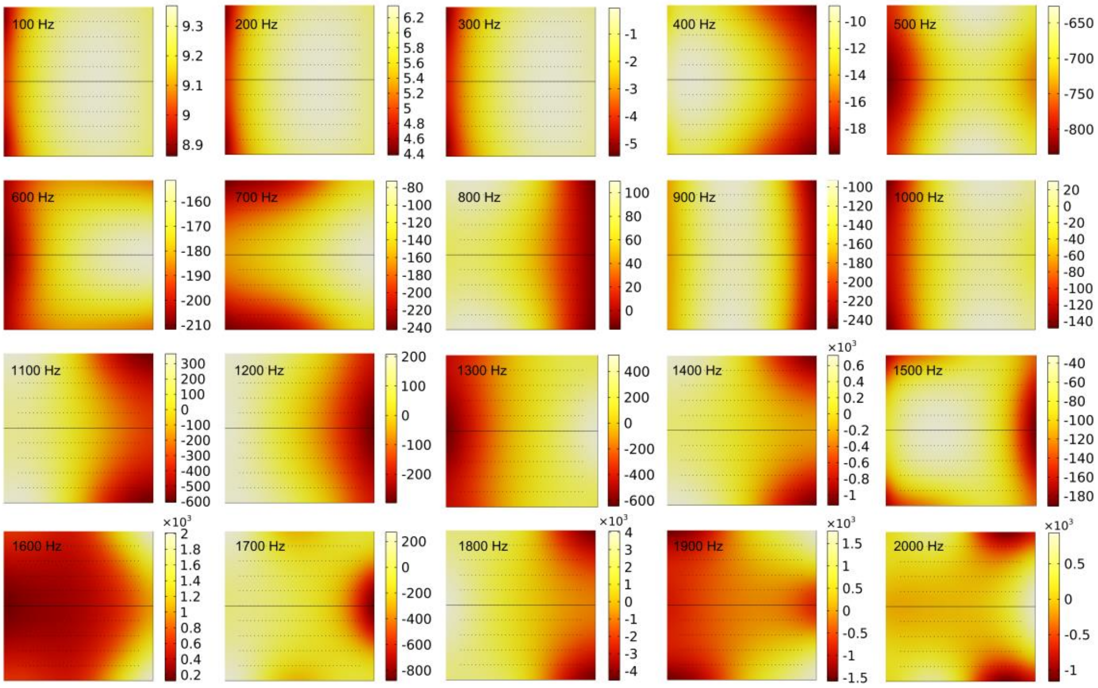

Supplementary Figure S1: COMSOL Simulation Results illustrating the acoustic pressure distribution across a range of frequency values from 100 Hz to 2 kHz. As observed in the figure, the distribution appears more stable at lower frequency values, whereas at higher frequencies, it becomes chaotic. Considering these results, a frequency value of 10 Hz has been selected for further investigation.

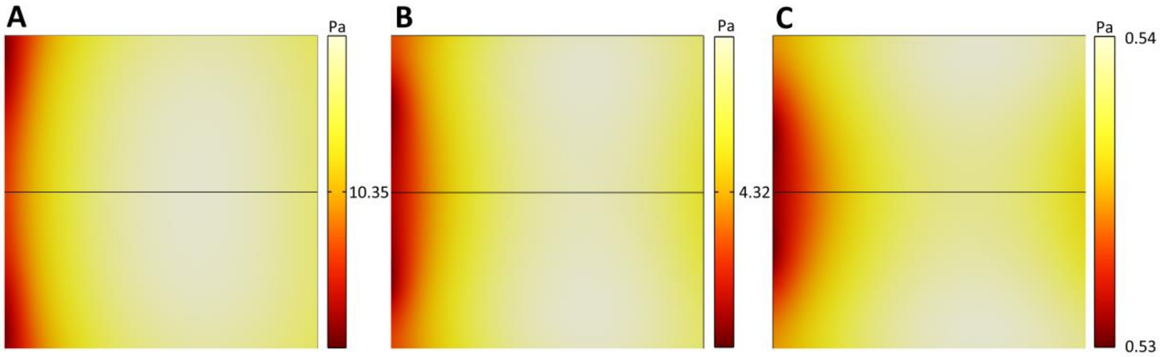

Supplementary Figure S2: COMSOL Simulation Results depicting the acoustic pressure distribution for square-shaped imaging areas with edge lengths of 10, 15, and 20 mm, respectively.
